# Arc accretion and crustal reworking from late Archean to Neoproterozoic in Northeast Brazil

**DOI:** 10.1038/s41598-020-64688-9

**Published:** 2020-05-12

**Authors:** Alanielson C. D. Ferreira, Elton L. Dantas, Reinhardt A. Fuck, Ingrid M. Nedel

**Affiliations:** Instituto de Geociências, Universidade de Brasília (UnB), 70910-900 Brasília, DF Brazil

**Keywords:** Precambrian geology, Tectonics

## Abstract

New systematic Nd isotope and U-Pb geochronology data were applied to Precambrian rocks of northeastern Brazil to produce a crustal-age distribution map for a small basement inlier (1,500 km^2^). The results support episodic crustal growth with five short periods of crustal formation at ca. 2.9 Ga, 2.65 Ga, 2.25 Ga, 2.0 Ga, and 0.6 Ga. Based on the frequency histogram of U-Pb zircon ages and Nd isotopic data, we suggest that about 60% of the continental crust was formed during the Archean between 2.9 Ga and 2.65 Ga. The remaining 40% of crust was generated during the Rhyacian to Neoproterozoic (~2.0–0.6 Ga). This overall continental growth is manifested by accretionary processes that involved successive accretions surrounding an older core, becoming younger toward the margin. Strikingly, this repetitive history of terrane accretion show a change from lithospheric peeling dominated accretionary setting during the late Archean to a more, modern-day akin style of arc-accretion during the Proterozoic. Similar tectonic processes are observed only in large continental areas (>1,000,000 km^2^) as in the North American continent basement and in the Amazonian Craton.

## Introduction

Understanding the evolution of the continental crust is a challenge due to the diversity of geological environments where it forms and to the variety of reworking processes it may have undergone throughout the geological time. Chelogenic cycles^1^, terrane accretion^2^, or continental collision are among fundamental processes that allow the preservation of the archives of crustal evolution^3–6^. Particularly, terrane accretion is one of the main processes for lateral continental growth through Earth’s history^6–9^.

The formation processes for the early Archean tonalite–trondhjemite–granodiorite (TTG) associations is incompatible with the Phanerozoic-style of subduction^10^. This initial TTG generation was through partial melting of hydrated low-Mg basaltic rocks within the base of a thickened basaltic crust^11,15^. However, the 3.2 Ga Mesoarchean to 2.3 Ga Paleoproterozoic continental crust may represent a transition period from an early non-plate tectonic mode to modern-style plate tectonics by accreted oceanic arcs and oceanic plateaus, mainly through ultrahigh-temperature processes^12–15^. Therefore, the preservation of Meso- to Neoarchean felsic continents may represent the initiation of plate tectonics in some form^15,16^. In this debate, the application of a geodynamic unifying model or the reconciliation of different models for the ancient continents generation is still in dispute^15–17^. However, it appears that there was a shift from the Archean continental crust produced by accretion and lithospheric peeling processes to Proterozoic continental crust generated by magmatic arcs^18–21^. At the center of this debate is the mechanical behavior of subsiding crust during the Archean and its lifetime, and how the transition to continental arcs and Phanerozoic-style subduction took place^18,21^. Some studies suggest long time scales (3.2 to 2.5 Ga) for a profound change in average crustal chemistry^22^. Gradual decrease in the rate of crust generation may be explained by the secular cooling of the mantle^23^, and the decline in crustal reworking may be explained by the “cratonization” of continental crust^4^.

Compositional diversity and complex evolution of the accretionary orogens are related to the plate boundary parallel migration, and orthogonal accretion of juvenile and reworked crustal segments^9^. In this context, Sm-Nd isotopes may provide a mean for determining (1) the crustal residence time^24,25^, (2) crustal reworking processes^26^, and (3) mantle mixing^27^. Therefore, Nd isotopes allow the characterization of protolith sources as a way to describe the geometry and direction of continental crust growth^24,28^.

In this study, we show evidence of continental growth via terrane accretion within the Campo Grande Block of the Borborema Province, NE Brazil. Using petrographic mapping, and spatial distribution of coupled U-Pb zircon ages and Sm-Nd isotopic data, we show that repetitive accretion of crustal terranes occurred within this area from the late Archean to the Neoproterozoic.

## Regional Geology

**Figure 1:**
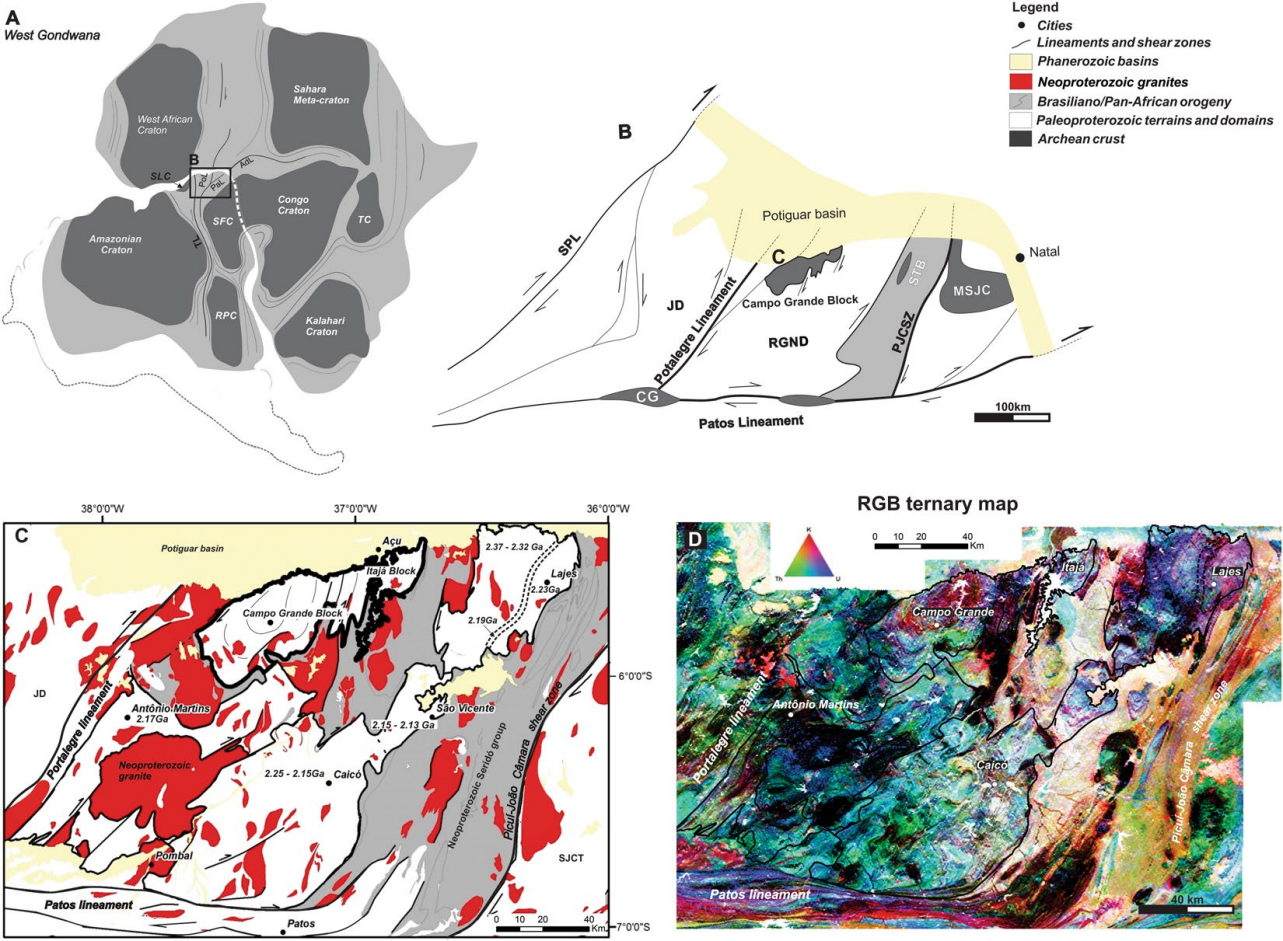
Regional geological setting. (**A**) Localization map of the Borborema Province in West Gondwana. (**B**) Geological map of the central portion of the Rio Grande do Norte domain. (**C**) U-Pb zircon age distribution and (**D**) ternary gamma-spectrometric map of the Caicó-São Vicente, Lajes, Antônio Martins and Campo Grande-Itajá regions in which the Rio Grande do Norte basement is exposed^29,31,36,44^. Note that the Campo Grande-Itajá area represents the unique basement dome in the Rio Grande do Norte domain (**D**). Legend: RPC - Rio de La Plata Craton, SFC - São Francisco Craton, SLC – São Luiz Craton, TC - Tanzania Craton. PoL - Portalegre Lineament, PJCSZ – Picuí-João Câmara shear zone, PaL - Patos Lineament, ADL - Adamaoua Lineament. JD - Jaguaribe domain, RGND - Rio Grande do Norte domain, and SJCM - São José do Campestre massif.

The Borborema Province is a Precambrian shield^29–33^ within the north-northeastern part of the South American continent^30,31^ (Fig. 1A). It is formed of discontinuous remnants of Archean crust, Paleoproterozoic migmatitic gneiss complexes, and Meso- to Neoproterozoic supracrustal rocks^29,31,32^. The Paleoproterozoic complexes comprise the 2.2-2.0 Ga gneiss-migmatite basement of Neoproterozoic supracrustal sequences and granite intrusions^34,36^. These high-grade gneisses and anatectic domes may be related to the 2.25-1.98 Ga Eburnian Orogeny^30,35,36^. The final configuration of the Borborema Province resulted from the diachronic convergence of the West African, Amazonian and São Francisco-Congo cratons during the Neoproterozoic Brasiliano/Pan-African orogeny^33,35^ (Fig. 1A).

The Rio Grande do Norte domain (RGND; Fig. 1B), the northeastern portion of the Borborema Province, is limited westwards by the NE-trending rectilinear Portalegre dextral strike-slip shear zone and by the Patos-Adamaoua EW-trending shear zone at the southern boundary^29,31,34^. Several shear zones represent local adjustments within each terrain, as well as divide the RGND into four high-grade migmatite-gneiss blocks (e.g., Caicó, Lajes, Antônio Martins and Campo Grande-Itajá; Fig. 1C, D). Zircon U-Pb ages indicate that Rhyacian (2.25 to 2.15 Ga) metamorphic high-K calc-alkaline magmatic rocks^37^ and supracrustal rocks form the basement of the Neoproterozoic Seridó Group^32^.

## Result and Discussion

### Geology

The Campo Grande Block is a small crustal fragment, 1,500 km^2^ in area, with dome to ellipsoidal geometry, SSW-NNE axis, exposed in the central portion of the Rio Grande do Norte domain, around Campo Grande town (Fig. 1B, C). The CGB consists of an Archean tonalitic to granitic migmatite complex and mafic-ultramafic rocks in the core, rimmed by Paleoproterozoic alkaline orthogneisses, surrounded by an outer rim of Neoproterozoic K-feldspar-rich granite intrusions (e.g., Caraúbas granite). The block shows intense deformation, with coaxial refolding, pervasive foliation, and north-northeast trending shear zone systems^38,39^. The Campo Grande-Itajá region represents a unique basement dome in the Rio Grande do Norte domain (Fig. 1D). The migmatites in the central area display higher Th and K concentrations (Fig. 1D), followed by an abrupt reduction of these elements in the inner rim orthogneiss, and again high contents in the outer rim granite, reflecting distinct geological compartments from west to east. In addition, based on integrated analysis of structural pattern, ternary gamma-spectrometric map (Fig. 1D) and thorium anomaly map, we suggest that shear zone systems define major terrane boundaries. The Portalegre Lineament corresponds to a 20–40 km wide shear zone that separates the Rio Grande do Norte and Jaguaribe domains (Fig. 1C). The Paraú Lineament divides the west part of the Rio Grande do Norte domain into the distinct eastern Itajá and western Campo Grande blocks.

The Campo Grande Block consists of migmatitic gneisses that display multiple phases of partial melting^38^. These migmatites comprise Archean tonalitic gneisses that contain granitic Proterozoic leucosomes and alkali granite dikes. The mafic-ultramafic rocks comprise amphibolites and pyroxenites that are present as boudinaged bodies within the Archean migmatitic complex, which are further oriented parallel to the leucosomal layers of the host migmatites^39^. The overall outcrop pattern suggests that these mafic-ultramafic rocks were originally emplaced as dykes, intruding the host migmatitic gneisses. The ultramafic pyroxenites show relict cumulate texture, and re-equilibration to cummingtonite-grunerite-rich rocks, with varying proportions of chlorite, serpentine and magnetite. Amphibolites comprise massive poikiloblastic garnet and granoblastic amphibole with variable proportions of plagioclase + clinopyroxene in symplectitic texture, typical of retrograded high-pressure rocks^39^. The Itajá Block is composed of Paleoproterozoic K-feldspar-rich orthogneiss, and wehrlite intrusions that occur as elongated boudins (<100 m) in the host orthogneiss; minor amphibolite and supracrustal rocks also appear. Neoproterozoic pegmatite and alkaline granite intrusions make up almost 20–30% of both blocks.

### Spatial Pattern of Ages based on the Nd Evidence for Diachronous Crustal Accretion

**Figure 2:**
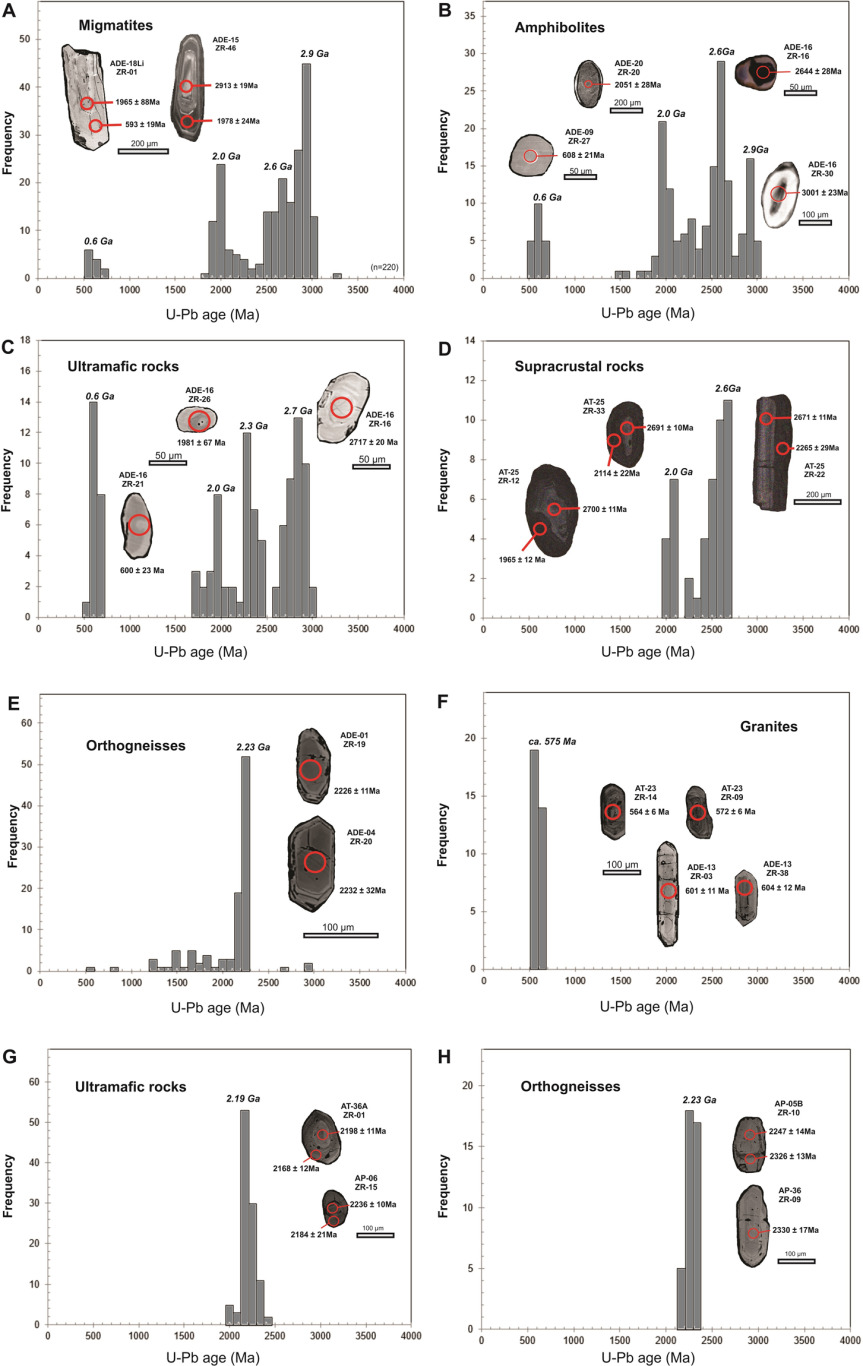
(**A** – **F**) Histograms of U-Pb zircon ages of the Campo Grande Block separated by different rock type with cathodoluminescence images of representative zircon grains (Data from supplementary Table). (**G**,**H**) Histograms of U-Pb zircon ages of the Itajá Block separated by different rock type with cathodoluminescence images of representative zircon grains (Data from supplementary Table 2).

The evolution of the Campo Grande Block involves at least seven thermal-tectonic events (Supplementary Table 1). The first magmatic event remains recorded in 2.98 to 2.91 Ga old tonalitic paleosome (Fig. 2A), which constitutes the central core of the block. All zircon crystals from tonalite samples are prismatic (100 to 300 μm), with Th/U ratios from 0.125 to 0.583 and internal zonation (Fig. 2A), all typical features of magmatic crystals^40^. The 2.9 Ga calc-alkaline magma represents a rare record of this age^41^, particularly in West Gondwana^42,43^. Inherited zircon cores of 3311 ± 52 Ma suggest a Paleoarchean crust as protolith source for the 2.9 Ga magmatism. The second partial melting event is represented by 2.65 Ga alkaline leucosome (e.g., ADE-23 sample) with thick K-feldspar-rich layers from the central portion of the strongly migmatized Archean core. Forty-four prismatic zircon crystals from this sample yield a Neoarchean Discordia age of 2651 ± 19 Ma. The 2.0 Ga and ca. 600 Ma zircon cores and rims are recorded in the migmatites. For example, the ADE-12 granitic migmatite sample yielded only 2.0 Ga prismatic zircon grains, while most of ca. 600 Ma Neoproterozoic ages are obtained in the overgrowth rims from the Archean migmatite zircon cores.

The clinopyroxene-garnet amphibolite lenses show the same 2.69-2.65 Ga age range, interpreted as the crystallization age of the protolith, also based on internal zonation, morphology and high Th/U ratio in zircon cores (Fig. 2B). The well-rounded (50 to 100 μm) zircon grains from amphibolite samples show zonation from core to rim, with well-defined rims, showing low to very bright luminescence (Fig. 2B), therefore indicating subsequent resorption and recrystallization^40^. The amphibolite samples also have 2.0 Ga well-rounded zircon crystals with zoned cores followed by outermost CL-bright overgrowths (Fig. 2B) possibly due to a subsequent event. The ca. 600 Ma Neoproterozoic homogeneous zircon grains, without internal zonation (Fig. 2B), are also recorded in the amphibolites. This confirms that the 2.65 Ga tholeiitic intrusions were subsequently torn apart during 2.0 Ga and 600 Ma tectonothermal events and are now present as isolated boudins. The 2.9 Ga inherited zircon grains were captured or assimilated by 2.65 Ga tholeiitic magma during its ascent and emplacement in the 2.9 Ga host tonalite basement^39^. Furthermore, as there are no fine-grained felsic veins intruding the amphibolite, it is unlikely that the 2.65 Ga zircon grains obtained in the mafic lenses came from other sources^39^.

The ultramafic pyroxenite lenses contain 2.7-2.6 Ga Archean and 2.3 Ga Paleoproterozoic zircon crystals with distinct morphological features like oscillatory zoning (Fig. 2C), typical of magmatic zircon^39^. Furthermore, pyroxenite samples present 2.0 Ga and ca. 600 Ma zircon grains that show varying degrees of rounding or absorbed borders (Fig. 2C), similar to features described in zircon grains from ultramafic rocks in high-grade metamorphic terrains^44,45^. The supracrustal rocks, such as garnet-biotite gneiss (AT-23 sample), bear 2.7-2.6 Ga zircon cores, suggesting that Neoarchean tonalite and tholeiitic rocks were the main provenance (Fig. 2D). The majority of these Archean zircon cores from the supracrustal sample display 2.2 to 2.0 Ga overgrowth rims (Fig. 2D). Besides, a few 2.46 to 2.44 Ga old zircon grains obtained in the Archean tonalite and supracrustal rocks suggest restricted Siderian magmatism.

In the eastern portion of the Campo Grande Block, the K-feldspar-rich alkali granite magmatism of 2.23-2.18 Ga Rhyacian age generated a large volume of magmatic rocks (Fig. 2E). Lastly, the K-feldspar-rich (20–30%) granitic plutons make up the western limit of the study area. The feldspar crystals develop a strong foliation parallel to the transcurrent shear zone. Neoproterozoic granites emplaced along the Portalegre shear zone (ADE-13 sample) have elongated prismatic zircon grains (3:1) that yield a crystallization age of 604 ± 12 Ma (Fig. 2F). On the other hand, granitic intrusions sampled in the central portion of the Campo Grande Block (e.g., AT-23 sample) show prismatic zircon crystals (2:1) crystallized at 566 Ma (Supplementary Table 1; Fig. 2F).

**Figure 3:**
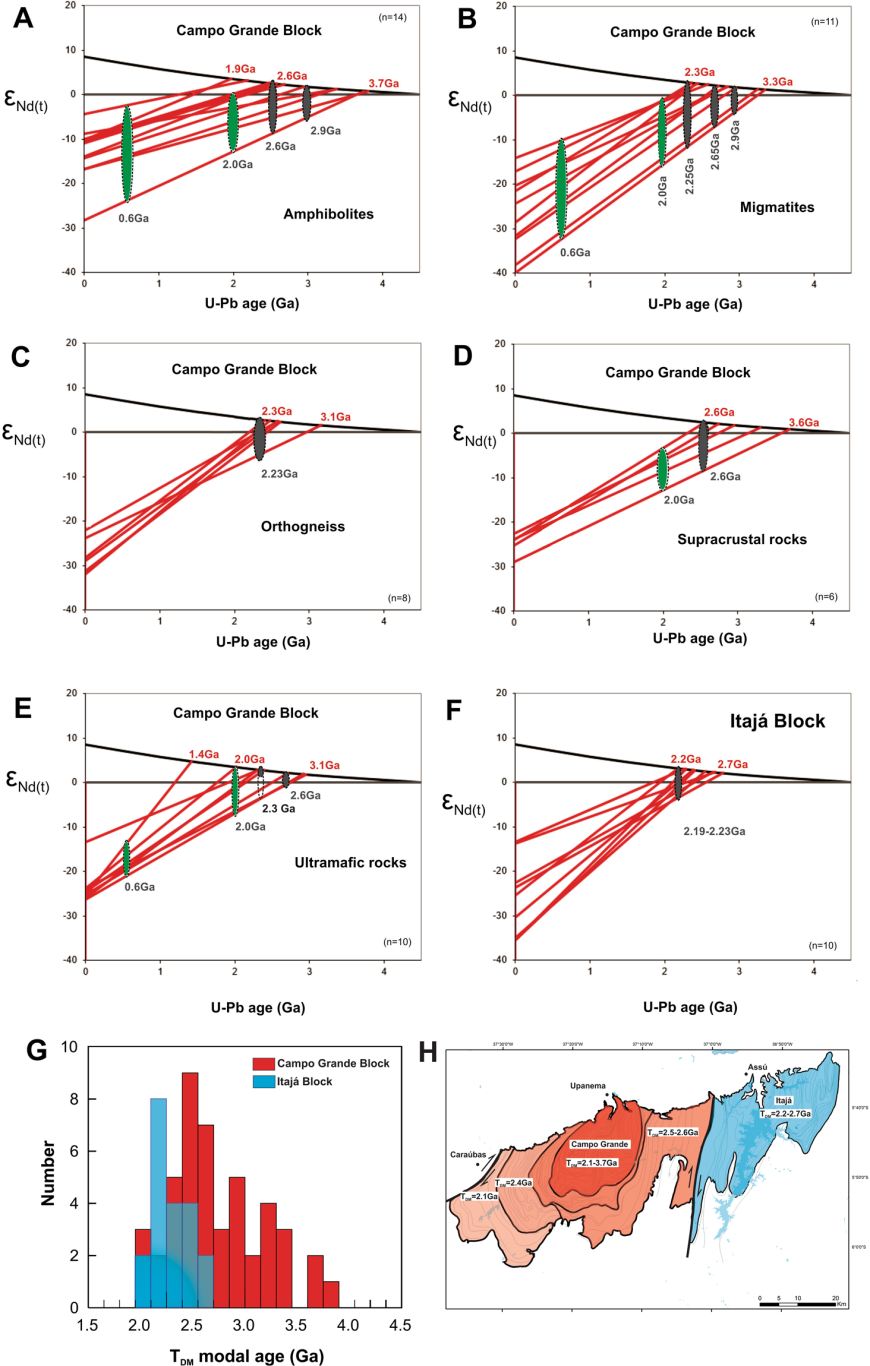
(**A**–**F**) εNd_(t)_ versus U-Pb zircon age from the major rock-types for the Campo Grande and Itajá blocks (Data from Table 1), Northeast Brazil. Gray ellipse - magmatic age, green ellipse - metamorphic age. (**G**) Histogram of TDM model age for the Campo Grande and Itajá blocks. (**H**) Schematic model of continental accretion for the Campo Grande and Itajá blocks.

The T_DM_ model ages and ε_Nd(t)_ values of migmatite samples support a complex history for the study area (Table 1, and Fig. 3A to H). The 2.9 Ga tonalitic migmatite displays positive and negative ε_Nd(t)_ values of -3.9 to +4.8 with T_DM_ model ages between 3.3 and 2.7 Ga, suggesting juvenile sources and crustal reworking at 2.9 Ga (Fig. 3B). All these Archean rocks are concentrated in the core of the structural dome of the Campo Grande Block. The 2.65 Ga and 2.0 Ga old alkaline granitic migmatites have negative ε_Nd(t)_ values (−5.47 to −2.74) and younger T_DM_ model ages between 2.8 and 2.4 Ga. The 2.65 Ga old amphibolites display negative ε_Nd(t)_ values (−1.03 to −7.97) with older T_DM_ model ages (3.7 to 3.3 Ga) and positive ε_Nd(t)_ values (+1.97 to +8.17) with younger T_DM_ model ages of 2.0 to 2.65 Ga, supporting a Neoarchean juvenile source (Fig. 3A) and contamination of crustal material.

**Table 1:** Nd isotope data and U-Pb zircon age for the Campo Grande and Itajá blocks.

Block	Sample	UTM X	UTM Y	Rock	Sm (ppm)	Nd (ppm)	^147^Sm/^144^Nd	^143^Nd/^144^Nd ± 2SE	ε_Nd(0)_	T_DM_(Ga)	U-Pb zircon age (Ga)						(Ma)
Campo Grande	ADE-10	679434	9355088	Tonalic migmatite	5.56	33.21	0.101138	0.510677 + /-8	−38.25	3.22	2.92						
Campo Grande	ADE-23	688721	9359183	Alkaline migmatite	17.43	93.43	0.112748	0.511325 + /-4	−25.60	2.59		2.65	2.46	2.23	2.13	1.93	
Campo Grande	ADE-08	692208	9353364	Granitic migmatite	2.31	10.51	0.133022	0.511755 + /-3	−17.22	2.42							
Campo Grande	At-06	695271	9358884	Granitic migmatite	30.87	138.08	0.135130	0.511592 + /-10	−20.41	2.82							
Campo Grande	At-02	696541	9353516	Granitic migmatite	4.60	19.02	0.146310	0.511909 + /-11	−14.23	2.56		2.71	2.4			1.99	
Campo Grande	At-13b	683328	9360094	Granitic migmatite	4.66	22.82	0.123469	0.511388 + /-10	−24.38	2.80							
Campo Grande	At-28	691307	9368206	Tonalic migmatite	3.17	19.14	0.100057	0.510589 + /-19	−39.97	3.31							
Campo Grande	ADE-15	691730	9370171	Tonalic migmatite	9.09	55.32	0.099317	0.511019 + /-1	−31.57	2.69	2.91					1.96	611
Campo Grande	ADE-18Li	683321	9360722	Alkaline migmatite	1.95	6.96	0.169067	0.511395 + /-14	−24.24		2.91			2.18		1.96	568
Campo Grande	ADE-12L	672348	9357353	Granitic migmatite	3.52	24.36	0.087379	0.510997 + /-11	−32.01	2.46						1.95	
Campo Grande	ADE-18P	683347	9364136	Tonalic migmatite	4.86	27.78	0.105862	0.510976 + /-8	−32.42	2.93	2.98						
Campo Grande	ADE-09	679495	9355415	Amphibolite	3.30	12.75	0.156692	0.512121 + /-3	−10.09	2.46		2.69				2.0	593
Campo Grande	ADE-16	687186	9367246	Amphibolite	4.33	16.95	0.154564	0.511773 + /-8	−16.88	3.33	3.01	2.65					593
Campo Grande	ADE-20	685014	9362028	Amphibolite	2.23	9.50	0.141955	0.512116 + /-5	−10.19	1.95							599
Campo Grande	ADE–24 A	687678	9361941	Amphibolite	4.50	16.94	0.160752	0.511780 + /-20	−16.74	3.75						2.0	
Campo Grande	ADE-24B	687678	9361941	Amphibolite	4.24	16.43	0.156052	0.512077 + /-2	−10.95	2.55							
Campo Grande	ADE-29	682062	9359773	Amphibolite	3.57	12.70	0.170028	0.512416 + /-13	−4.32	2.17	2.99	2.66					589
Campo Grande	At-10	694967	9361572	Amphibolite	5.90	24.53	0.145492	0.511922 + /-19	−13.97	2.50							
Campo Grande	At-14a	683321	9360722	Amphibolite	4.45	19.78	0.135880	0.511189 + /-4	−28.27	3.70							
Campo Grande	At-16	682499	9360150	Amphibolite	5.34	20.71	0.155846	0.512099 + /-12	−10.51	2.48							
Campo Grande	At-24	687154	9358978	Amphibolite	4.76	18.70	0.153968	0.511907 + /-6	−14.25	2.92							
Campo Grande	At-26	685121	9359016	Amphibolite	3.60	12.53	0.173462	0.512187 + /-12	−8.81	3.34							
Campo Grande	At-32	683195	9351556	Amphibolite	3.98	14.96	0.160949	0.512125 + /-15	−10.00	2.66							
Campo Grande	AP-10	689429	9365786	Amphibolite	5.31	19.65	0.163452	0.512011 + /-4	−12.23	3.19							
Campo Grande	AP-17	683241	9360032	Amphibolite	9.59	39.16	0.148096	0.512104 + /-3	−10.42	2.17							614
Campo Grande	ADE-01	719922	9374303	Orthogneiss	14.38	64.85	0.134007	0.511418 + /-8	−23.80	3.13				2.23			
Campo Grande	ADE-03	710259	9362444	Orthogneiss	6.09	36.57	0.100681	0.511149 + /-9	−29.05	2.55	2.96	2.64		2.19			640
Campo Grande	ADE-04	698901	9351772	Orthogneiss	5.01	31.62	0.095842	0.511032 + /-17	−31.33	2.59				2.23			
Campo Grande	ADE-06	690941	9347540	Orthogneiss	4.94	24.38	0.122628	0.511509 + /-6	−22.02	2.56				2.22			
Campo Grande	ADE-14	680057	9353112	Orthogneiss	5.05	32.01	0.095451	0.511166 + /-4	−28.72	2.41				2.23	2.15	1.98	
Campo Grande	AT-23	690837	9359006	Granite	26.93	169.75	0.095909	0.511189 + /-13	−28.27	2.39							566
Campo Grande	ADE-13	666358	9360156	Granite	12.48	70.90	0.106444	0.511554 + /-9	−21.15	2.10							603
Campo Grande	ADE-27	686696	9361990	Supracrustal	1.68	8.59	0.118218	0.511341 + /-10	−25.30	2.72							
Campo Grande	At-22	685228	9366806	supracrustal	4.46	20.18	0.133481	0.511153 + /-20	−28.98	3.65							
Campo Grande	At-25	685737	9359296	supracrustal	4.55	20.10	0.136809	0.511411 + /-8	−23.94	3.27		2.65	2.46	2.25	2.11	2.03	
Campo Grande	AP-12	688179	9362230	Supracrustal	1.67	7.66	0.131808	0.511478 + /-14	−22.63	2.92							
Campo Grande	AP-16	682735	9358176	Supracrustal	21.45	112.19	0.115598	0.511401 + /-3	−24.13	2.55							
Campo Grande	AP-18B	686983	9361480	Supracrustal	0.56	2.64	0.129098	0.511321 + /-11	−25.69	3.12							
Campo Grande	ADE-17	682759	9366783	Ultramafic	1.87	8.00	0.141493	0.511950 + /-18	−13.41	2.29							
Campo Grande	ADE-22	686715	9362241	Ultramafic	1.78	35.40	0.030348	0.511331 + /-2	−25.50	1.42							
Campo Grande	ADE-25A	687089	9362507	Ultramafic	0.69	3.58	0.117281	0.511341 + /-9	−25.29	2.69							
Campo Grande	ADE-25B	687089	9362507	Ultramafic							2.83		2.44	2.25		1.99	
Campo Grande	ADE-26A	686780	9362179	Ultramafic	1.00	4.71	0.128430	0.511413 + /-13	−23.90	2.92	2.95	2.68				1.99	600
Campo Grande	ADE-26C	686780	9362179	Ultramafic	0.68	3.40	0.121110	0.511288 + /-13	−26.33	2.90							
Campo Grande	ADE-28A	686279	9361529	Ultramafic	3.74	18.82	0.120027	0.511076 + /-43	−30.47	3.22							627
Campo Grande	AP-18A	686983	9361480	Ultramafic	2.28	13.99	0.098428	0.511302 + /-4	−26.06	2.29							
Campo Grande	AP-09	687408	9350474	Ultramafic	3.78	19.70	0.115932	0.511536 + /-4	−21.51	2.34				2.33			
Campo Grande	At -09	695051	9361072	ultramafic	6.51	36.71	0.107176	0.511378 + /-6	−24.58	2.37							
Campo Grande	AP-22	683311	9354674	Ultramafic	3.56	23.80	0.090438	0.511410 + /-4	−23.94	2.01	2.87	2.74					594
Itajá	ADE 31	743726	9380744	Ultramafic	2.821	12.212	0.1396	0.511946 + /-6	−13.50	2.25							
Itajá	AT-36A	749233	9369998	Ultramafic	0.711	2.874	0.1496	0.511928 + /-9	−13.86	2.66				2.19			
Itajá	AP-06	749296	9370562	Ultramafic	0.626	4.046\	0.0936	0.511330 + /-6	−25.51	2.16				2.23			
Itajá	AP-07A	750140	9372398	Ultramafic	2.098	10.244	0.1238	0.511419 + /-4	−23.77	2.75				2.19			
Itajá	AP-24A	749348	9379584	Ultramafic	3.714	34.332	0.0654	0.510820 + /-2	−35.47	2.27							
Itajá	AP-24B	749348	9379584	Ultramafic	4.103	34.332	0.0722	0.510819 + /-3	−35.49	2.39							
Itajá	AP-23A	750245	9377268	Ultramafic	2.704	14.141	0.1156	0.511479 + /-5	−22.61	2.42				2.29			
Itajá	AP-23B	750245	9377268	Orthogneiss	2.876	14.141	0.1229	0.511479 + /-5	−22.61	2.62							
Itajá	AP-05B	748379	9367658	Orthogneiss	13.904	103.507	0.0812	0.510843 + /-6	−35.01	2.52				2.23			
Itajá	PC-36	756328	9380004	Orthogneiss	3.675	25.248	0.0880	0.511081 + /-3	−30.37	2.37				2.32

The pyroxenites display heterogeneous Nd isotopic data (Table 1). The 2.6 Ga old pyroxenite samples display positive and negative ε_Nd(t)_ values with T_DM_ model ages between 2.6 and 3.2 Ga, whereas 2.3 Ga old pyroxenites show positive ε_Nd(t)_ values with restrict T_DM_ model ages of 2.29-2.37 Ga (Fig. 3E). The younger T_DM_ model ages of 1.4 and 2.0 Ga with strongly negative ε_Nd(t)_ values may suggest metamorphic alteration in the Sm-Nd isotopic system during Proterozoic times (Figs. 3E, 2C). Furthermore, we suggest that the negative values of ε_Nd(t)_ and older T_DM_ for the 2.65 Ga ultramafic rocks may reflect enriched sources or crustal assimilation. The supracrustal protoliths have T_DM_ model ages of 3.6 to 2.6 Ga with positive to negative ε_Nd(t)_ values for the 2.65 Ga crystallization age and negative ε_Nd(t)_ values (Fig. 3D) during Paleoproterozoic events. The 2.2 Ga K-feldspar-bearing augen orthogneisses display a Nd isotopic signature characterized by negative (-8.0) to positive (+5.0) ε_Nd(t)_ values and T_DM_ between 2.3 and 3.1 Ga (Fig. 3C), indicating a Rhyacian calc-alkaline magmatism with crustal reworking and juvenile sources contributions. Lastly, the Neoproterozoic granites present strongly negative ε_Nd(t)_ values (-20.57 and -14.25) with relatively younger T_DM_ model ages of 2.10 and 2.39 Ga (Table 1).

T_DM_ model ages and ε_Nd(t)_ values support a complex history for the Campo Grande Block (Fig. 3A–H). Whole-rock Nd isotope results indicate that the isotope system preserved the protolith source signature despite of crustal reworking and high-grade metamorphic events that affected the Archean core. Paleoproterozoic ages appear in the 1.95 Ga granitic leucosome generation and 2.0 Ga metamorphic overgrowth zircon rims on Neoarchean zircon cores from the ultramafic and supracrustal protolith rocks inside the Archean core. The Rhyacian orthogneisses from the eastern portion and 2.0 Ga granitic leucosome from the Archean central portions display similar T_DM_ model ages and ε_Nd(t)_, meaning that both K-feldspar-rich alkaline magmatism and crustal anatexis have similar sources. Nevertheless, crustal reworking was intense in the eastern block area, practically obliterating the Archean protolith record. A second high-grade metamorphic event - the seventh recorded event – is indicated by 614-593 Ma old zircon grains and rims around the Archean zircon cores from the amphibolite samples^39^. Moreover, 604 Ma old K-feldspar-rich granitic intrusions and 566 Ma pegmatite veins suggest a more restricted Neoproterozoic partial melting when compared to the large volume of neosome generated during the Rhyacian. The Neoproterozoic granite intrusions and alkaline leucosome samples have strongly negative ε_Nd(t)_ values (−20.57 and −14.25) and relatively younger T_DM_ ages of 2.10 and 2.39 Ga. These Nd isotope results suggest that the Paleoproterozoic crust is the main protolith source for the Neoproterozoic alkali granitic magmatism. That is, on the outermost overgrowths of the Archean dome the reworking process is dominant when compared to the core (Fig. 3G). The progressive decrease in T_DM_ model ages from the core (3.7 Ga) towards the margins (2.1 Ga) of the block, integrated with structural, thorium anomaly map, and U-Pb zircon age patterns suggest accretionary processes for the continental growth (Fig. 3H). Thus, Nd isotope evolution reflects the crustal growth from the Archean core protolith, following extensive Paleoprotezoic juvenile accretion and reworking, as well as Neoproterozoic crustal magmatism at the outer rim.

In contrast, the Itajá Block only records two events of magma generation (Supplementary Table 2). The first event is represented by orthogneisses that were formed at 2.23 Ga (Fig. 2H), displaying negative to weakly positive ε_Nd(t)_ values (Fig. 3F) and T_DM_ model ages between 2.2 and 2.7 Ga (Table 1). Clinopyroxenites and wehrlites, crystallized at 2.19 Ga (Fig. 2G), with positive ε_Nd_(t) values (Fig. 3F), intruded these orthogneisses, indicating juvenile tholeiitic magmatism. Therefore, alkali granitic and ultramafic magmatism took place in a short time interval of ~40 Ma (2.23 to 2.19 Ga), similar to the reported events in the Lajes Block^44^, which is exposed 40 km eastwards, separated from the Itajá Block by the Neoproterozoic Seridó intracontinental fold belt (Fig. 1C,D). Furthermore, inherited zircon grains of Siderian age (ca. 2.32 Ga) are recorded in the host orthogneiss from the Itajá area. The intense Rhyacian reworking obliterated the possible older sources (Fig. 2F,G). Therefore, a genetic correlation with the Archean core of the Campo Grande Block is unclear (Fig. 3G). Nevertheless, it is indisputable that the protolith sources are dominantly Neoarchean, as suggested for the Lajes Block^45^.

### Crustal Reworking and Terrain Docking

**Figure 4:**
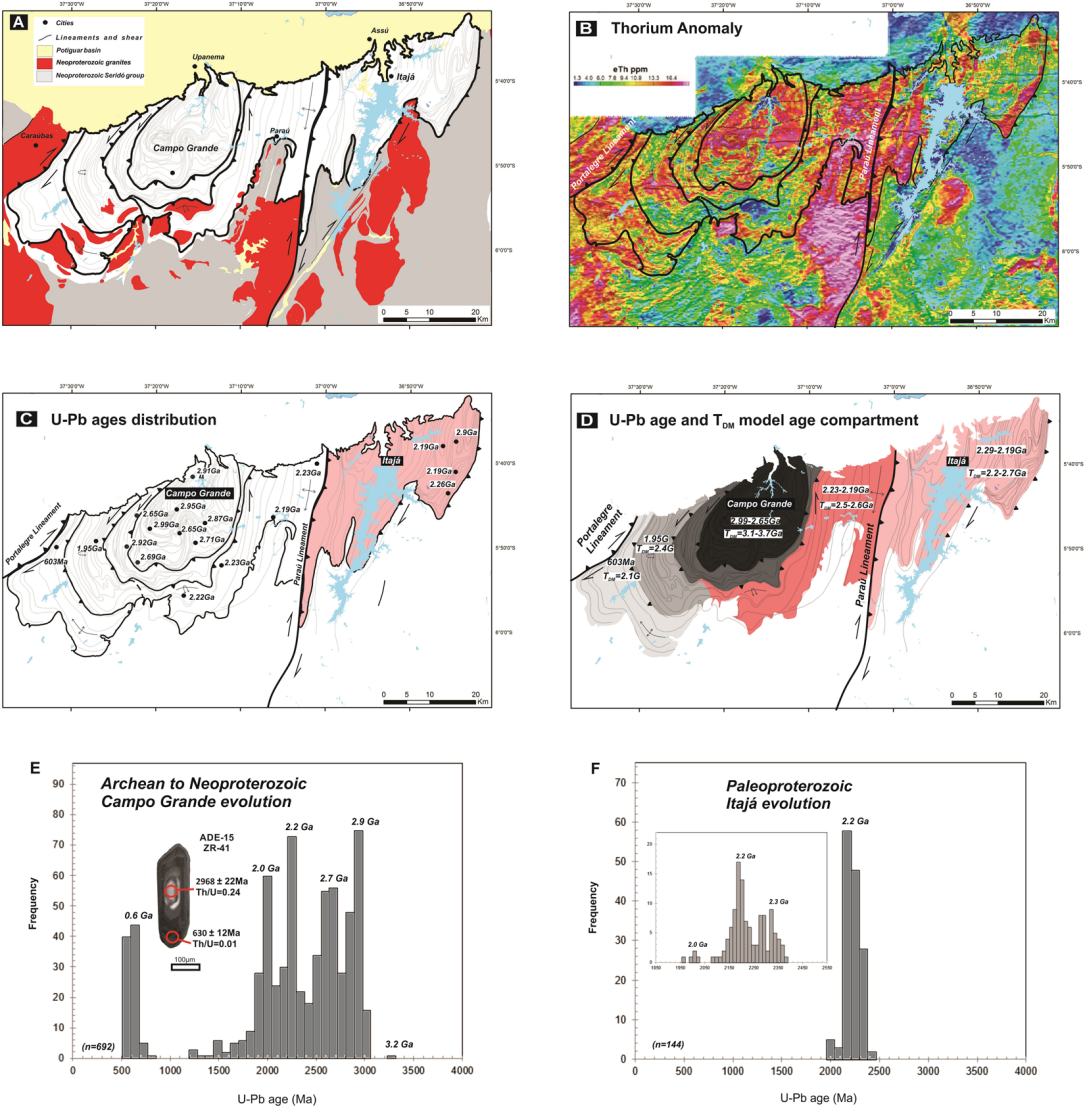
(**A**) Simplified geological and (**B**) Thorium anomaly map of the Campo Grande and Itajá blocks and adjacent areas. (**C**) U-Pb zircon age distribution of the Campo Grande and Itajá blocks. (**D**) Plot of crystallization ages and T_DM_ model ages of the Campo Grande and Itajá blocks (Data from Table 1). (**E**,**F**) Histograms of U-Pb zircon age of Campo Grande and Itajá blocks (Data from supplementary Table 1 and 2), Northeast Brazil.

The integration of all Nd isotope and U-Pb zircon age patterns allowed the establishment of limits and genetic correlations between the crustal fragments that form the Campo Grande and Itajá blocks (Fig. 4A–D). Our results support that 2.9 Ga and 2.7-2.6 Ga Archean crustal reworking and minor 2.2 Ga Paleoproterozoic juvenile mantle were the primary sources for the continental growth through accretionary mechanisms^5,15,16,19,45,46^. The first rim around the Archean core seems to engulf the core migmatites in a circular shape (Fig. 4A–D). This geometry is feasible via a 2.9 Ga domal fashion of tonalitic magmatism that engulfed the Archean core. However, the subsequent events may have occurred due to terrane accretionary mechanisms. Therefore, our results may indicate a change in the mechanism of continental evolution, namely dome formation at 2.9 Ga to terrane accretion starting at 2.7 Ga.

Based on the frequency histogram of the U-Pb zircon ages and the area mapped, it is suggested that at least 30–40% of the Campo Grande Block was already formed at 2.9 Ga (Fig. 3G, H). After 2.9 Ga, there was an increase in the rate of continental crust growth, probably due to subduction-like processes and peeling-off driven convergent settings^12,13,20^. Therefore, the accretionary orogenic collage derived from a complex diversity of protolith sources^47,48^, as described in this study (Fig. 4A–F). That is, the continental evolution is complex and includes several components of different scale, composition, and age^10,15,47,48^.

Thermal and compositional contrasts between continental and oceanic lithosphere lead to subsidence processes by plate tectonics^49,50^. The subsidence of oceanic crust allowed the efficient mechanical coupling of the microcontinents and remnant magmatic arcs in the orogenic wedge^2,3^. In this scenario, magmatic arc formation is probably the most important mechanism to maintain the continental crust reservoir^18,19^. Paleoproterozoic 2.25-2.18 Ga high-K calc-alkaline magmatism may represent a thermal weakening zone that allowed the reworking and juvenile magmatism^11,18^. In the Borborema Province, Paleoproterozoic arc magmatism represents a more significant period of crustal growth within the South American continent^19^, similar to the study area. Thus, terrain accretion and partial melting mainly in the root of the magmatic arc setting from 2.2 Ga promote the differentiation and growth of the continental crust^5,15,51^.

The preservation of the felsic continental block between 2.9 to 2.2 Ga in the Borborema Province may mark the transition and initiation of plate tectonics, implying a higher consumption of mafic crust during Proterozoic physical mechanisms of accretion compared to late Archean processes. One possibility would be crustal reworking via lower mafic crustal peeling-off (e.g. delamination) during continent-continent convergence^15,16^. Despite the significant increase in isotopic studies, late Archean reworking and recycling processes remain largely unknown^15,16^. Therefore, a different style of plate tectonics and subduction possibly occurred during the early Archean, with transitional physical mechanisms between the late Archean and the Phanerozoic-style. However, any model that calls upon fractionation of a single magmatic event or process to produce continental crust is unrealistic^51^.

## Conclusions

Nd isotope data and U-Pb geochronology within the distinct terrains provide constraints for the succession of magmatic and metamorphic phases that resulted in continental accretion of heterogeneous rocks from 2.9 Ga to ca. 566 Ma ago in northeast Brazil. These led to the assembly of the Rio Grande do Norte domain. The Campo Grande Block represents high-grade metamorphic terrains with multiple partial melting, meta-ultramafic, and metamafic lenses that record polyphase metamorphism, magmatism, and intense shearing. Our data bear evidence that the distribution and nature of the continental crust reflect the secondary processes of reworking. The age succession associated with the geochemical patterns of the Precambrian evolution of the Campo Grande Block highlights the importance of the accretionary dynamics for the continental growth. The accretionary process is cyclic and repeated in space and time, allowing the continental growth to start by Mesoarchean to Neoarchean crustal peeling-off driven lithospheric convergence to Proterozoic magmatic arc accretion. When the events ended at the Neoproterozoic (ca. 566 Ma), the Archean to Paleoproterozoic Campo Grande and Rhyacian Itajá complexes amalgamation in the center of West Gondwana was concluded.

## Methods

### Geological Mapping and Petrography

Geological mapping was undertaken in the Campo Grande area with the purpose of investigating the gneiss-migmatite complex. Geological mapping was supported by geochemical, geophysical and petrographic investigations. Systematic thin sections cut relative to foliation were obtained from representative samples from outcrops of migmatite, orthogneiss, ultramafic and supracrustal rocks. The petrography was done at the Microscopy Laboratory of the Institute of Geosciences of Universidade de Brasília (Brazil).

### U-Pb isotopes

Zircon grains from samples were separated by conventional procedures and magnetic separator after concentration by hand panning. U-Pb isotopic analyses were performed on zircon grains using a Thermo-Fisher Neptune High Resolution Multicollector Inductively Coupled Plasma Mass Spectrometer (HR-MC-ICP-MS) coupled with a Nd:YAG UP213 New Wave laser ablation system at the Laboratory of Geochronology of Universidade de Brasília. U-Pb analyses on zircon grains were carried out by the standard-sample bracketing method^52^, using the GJ-1 standard zircon^53^ in order to quantify the amount of ICP-MS fractionation. The tuned masses were 238, 207, 206, 204 and 202. The integration time was 1 second and the ablation time was 40 seconds. A 30 µm spot size was used and the laser setting was 10 Hz and 2-3 J/cm^2^. Two to four unknown grains were analyzed between GJ-1 analyses. ^206^Pb/^207^Pb and ^206^Pb/^238^U ratios were time corrected. The raw data were processed off-line and reduced using an Excel worksheet^54^. During the analytical sessions, the zircon standard 91500^55^ was also analyzed as an external standard.

Common ^204^Pb was monitored using the ^202^Hg and (^204^Hg + ^204^Pb) masses. Common Pb corrections were not done due to very low signals of ^204^Pb (<30 cps) and high ^206^Pb/^204^Pb ratios. Reported errors are propagated by quadratic addition [(2SD^2^ + 2SE2)1/2] (SD = standard deviation; SE = standard error) of external reproducibility and within-run precision. External reproducibility is represented by the standard deviation obtained from repeated analyses (~1.1% for ^207^Pb/^206^Pb and up to ~2% for ^206^Pb/^238^U) of the GJ-1 zircon standard during the analytical sessions, and the within-run precision is the standard error calculated for each analysis. Concordia diagrams (2σ error ellipses), probability density plots and weighted average ages were calculated using the Isoplot-3/Ex software^56^.

### Sm-Nd Isotopes

Sm–Nd isotopic analyses followed the method described by Gioia and Pimentel (2000)^57^ and were also carried out at the Geochronology Laboratory of Universidade de Brasília. Whole-rock powders (~50 mg) of 60 samples were mixed with ^149^Sm–^150^Nd spike solution and dissolved in Savillex Digestion Vessels. Sm and Nd extraction of whole-rock samples followed conventional cation exchange chromatography techniques, with Teflon columns containing LN-Spec resin (HDEHP – diethylhexil phosphoric acid supported on PTFE powder). Sm and Nd fractions were loaded on Re evaporation filaments of double filament assemblies, and the isotopic measurements were carried out on a multicollector TRITON thermal ionization mass spectrometer in static mode. Uncertainties of Sm/Nd and ^143^Nd/^144^Nd ratios were better than ±0.1% (2 σ standard error) and ±0.0015% (1σ), respectively, according to repeated analyses of the international rock standard BHVO-1. ^143^Nd/^144^Nd ratios were normalized to ^146^Nd/^144^Nd = 0.7219, and the decay constant used was 6.54 ×10^−12^. The T_DM_ values were calculated using the DePaolo (1981) model^24^.

## Supplementary information

Table 01.

Supplementary Table 01.

Supplementary Table 02.

## Data Availability

The authors Alanielson da C. D. Ferreira, Elton L. Dantas, Reinhardt A. Fuck, and Ingrid M. Nedel accept and declare the availability of data.
